# Basal Cell Carcinoma With Calcification: Case Report of Calcifying Basal Cell Carcinoma and Review of Calcinosis Cutis Associated With Basal Cell Carcinoma

**DOI:** 10.7759/cureus.12721

**Published:** 2021-01-15

**Authors:** Parnia Forouzan, Antoanella Calame, Nathan S Uebelhoer, Philip R Cohen

**Affiliations:** 1 Medicine, McGovern Medical School, University of Texas Health Science Center at Houston, Houston, USA; 2 Dermatology/Dermatopathology, Compass Dermatopathology, San Diego, USA; 3 Dermatology, Scripps Memorial Hospital, La Jolla, USA; 4 Dermatologic Surgery, San Diego Family Dermatology, National City, USA; 5 Dermatology, San Diego Family Dermatology, National City, USA

**Keywords:** basal, calcification, calcinosis, calcium, carcinoma, cell, cutis, histology, mammography, nodular

## Abstract

Basal cell carcinoma is the most common cutaneous neoplasm. Calcinosis cutis is the deposition of calcium within the dermis. An 80-year-old man presented with a pearly nodule on his left nasal ala; a shave biopsy confirmed the diagnosis of a nodular basal cell carcinoma with calcinosis cutis, which was removed with Mohs micrographic surgery. The incidence of basal cell carcinoma with calcinosis cutis as well as the classification, identification, and potential origin of calcium deposits in basal cell carcinoma are discussed. Basal cell carcinoma can be associated with calcinosis cutis; indeed, calcifying basal cell carcinoma has a calculated incidence of 14%. There are five categories of calcification in basal cell carcinoma. In addition, calcification observed in cancer-free initial sections of a suspected basal cell carcinoma may be a diagnostic clue that a neoplasm is present in deeper sections of the tissue specimen. Although nodular basal cell carcinoma has the greatest incidence (37%) of calcium deposition, infiltrative (29%) and micronodular (27%) basal cell carcinomas are also frequently associated with calcification; therefore, the presence of calcifying basal cell carcinoma may indicate a more aggressive tumor subtype. Basal cell carcinoma may also be suspected in the differential diagnosis of a superficial breast neoplasm in which calcification is observed in the dermis; in this situation, mammography has been an effective diagnostic approach for identifying the basal cell carcinoma with calcification. The pathogenesis of calcification in basal cell carcinoma remains to be definitively established; however, calcium-binding proteins found in poorly differentiated keratinocytes may contribute to the etiology of basal cell carcinoma with calcification. The treatment of basal cell carcinomas with calcinosis cutis is similar to that of non-calcifying basal cell carcinomas; it is based upon the histologic subtype, the size, and the location of the tumor.

## Introduction

Basal cell carcinoma is the most common cutaneous neoplasm. There are several clinical and pathological subtypes. The treatment varies depending on the location and subtype of basal cell carcinoma and includes destruction (such as with cryotherapy and electrodessication), Mohs micrographic surgery, radiotherapy, and topical therapy (such as with 5-fluorouracil or imiquimod) [1].

Calcinosis cutis is the deposition of calcium salts within the dermis. Calcinosis cutis can be readily identified on tissue specimens stained with hematoxylin and eosin. If necessary, calcium deposition can be confirmed using Von Kossa staining. There are four classifications of calcinosis cutis: dystrophic, iatrogenic, idiopathic, and metastatic [2,3].

An 80-year-old man whose basal cell carcinoma demonstrated calcinosis cutis is described. He presented with a nodular basal cell carcinoma on his nasal ala, which required three stages of Mohs micrographic surgery to remove. The characteristics of basal cell carcinoma that have associated calcinosis cutis are summarized.

## Case presentation

**Figure 1:**
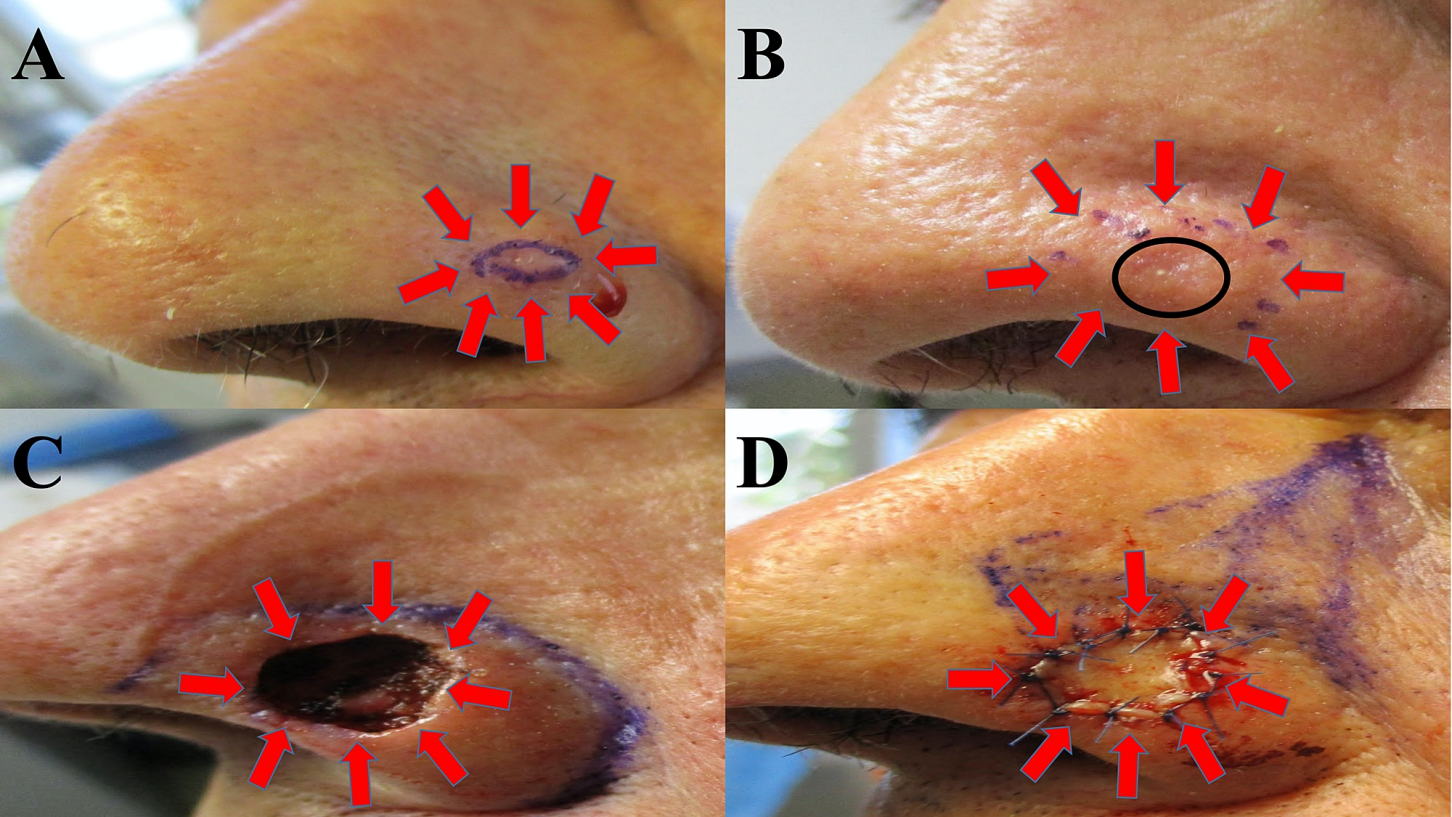
Clinical presentation of calcifying basal cell carcinoma and removal with Mohs microscopically controlled surgery An 80-year-old man presented with a nodule on his left nasal ala (A); the tumor is surrounded by red arrows, and the biopsy site is the skin within the purple oval. The lesion site was injected with epinephrine-containing anesthetic before the shave biopsy, accounting for whitening of the surrounding tissue. The biopsy site (B) was healed at four weeks follow-up (black circle), and Mohs surgery was scheduled to excise the nodular basal cell carcinoma with calcinosis cutis (surrounded by red arrows). After three stages of Mohs micrographic surgery, clear tumor margins were obtained; the resulting skin defect on the left nasal ala is surrounded by red arrows (C). A skin-advancing flap was initially planned (purple lines demarcating the planned incisions) to close the nasal defect (D); however, a full thickness skin graft (surrounded by red arrows) from posterior auricular skin was used instead.

An 80-year-old man with a history of non-melanoma skin cancer presented for a scheduled skin check. Cutaneous examination showed a five-millimeter nodule on his left nasal ala (Figure 1). A shave biopsy was performed.

**Figure 2:**
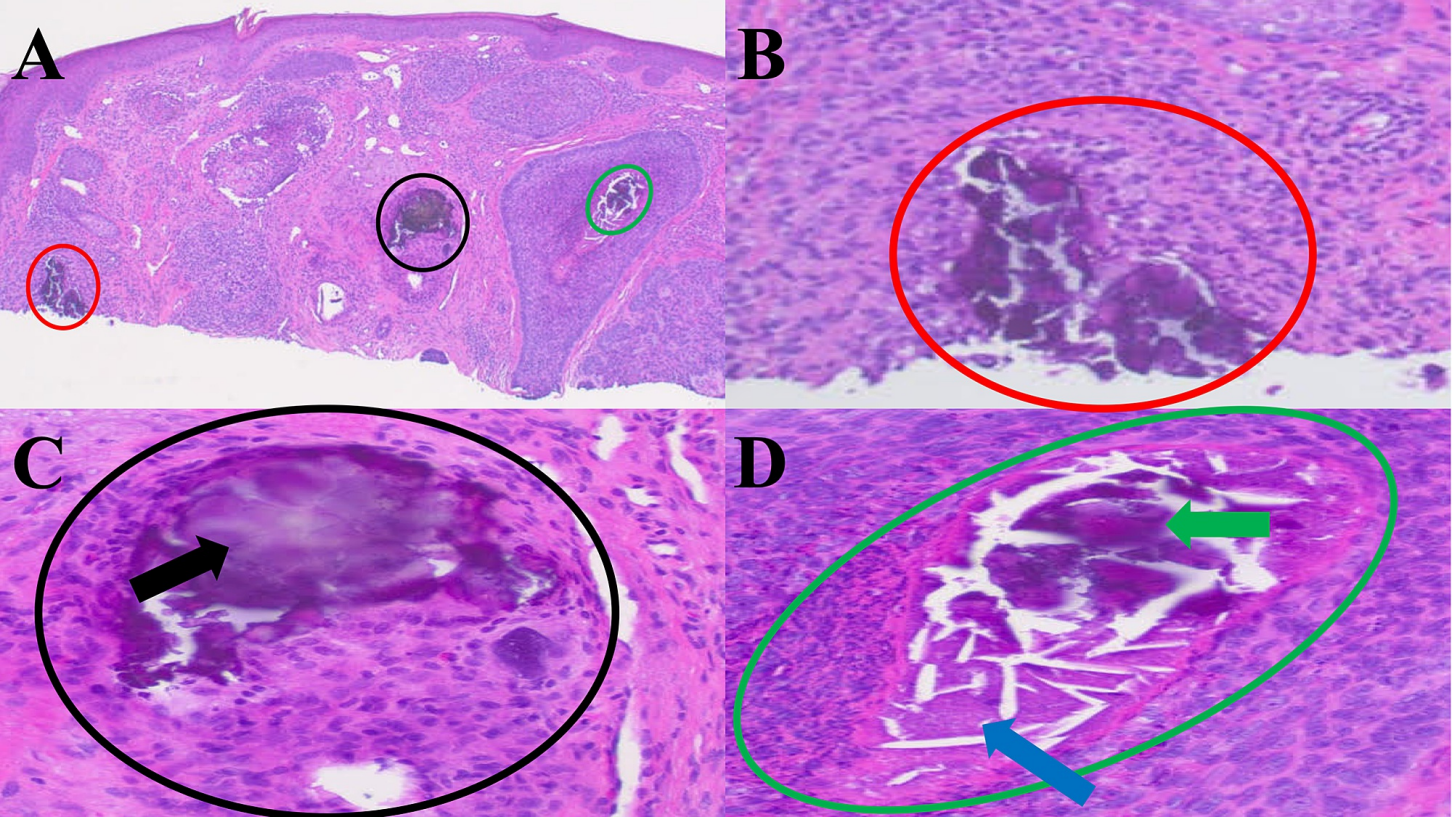
Histologic sections of a nodular basal cell with three areas of calcification The biopsy tissue specimen was bisected. One half of the bisected specimen shows three areas of calcification within the dermis (red, black, and green circles) associated with areas of basal cell carcinoma (A). A closer view (B) shows type 4 basal cell carcinoma-associated calcification free in the adjacent dermis (red circle). A closer view (C) shows type 5 basal cell carcinoma-associated calcification (black arrow) with calcium in a hair follicle (black circle). A closer view (D) shows a type 3 basal cell carcinoma-associated calcification (green arrow) with calcium in an area of necrosis (blue arrow) within an aggregate of basal cell carcinoma (green circle).

**Figure 3:**
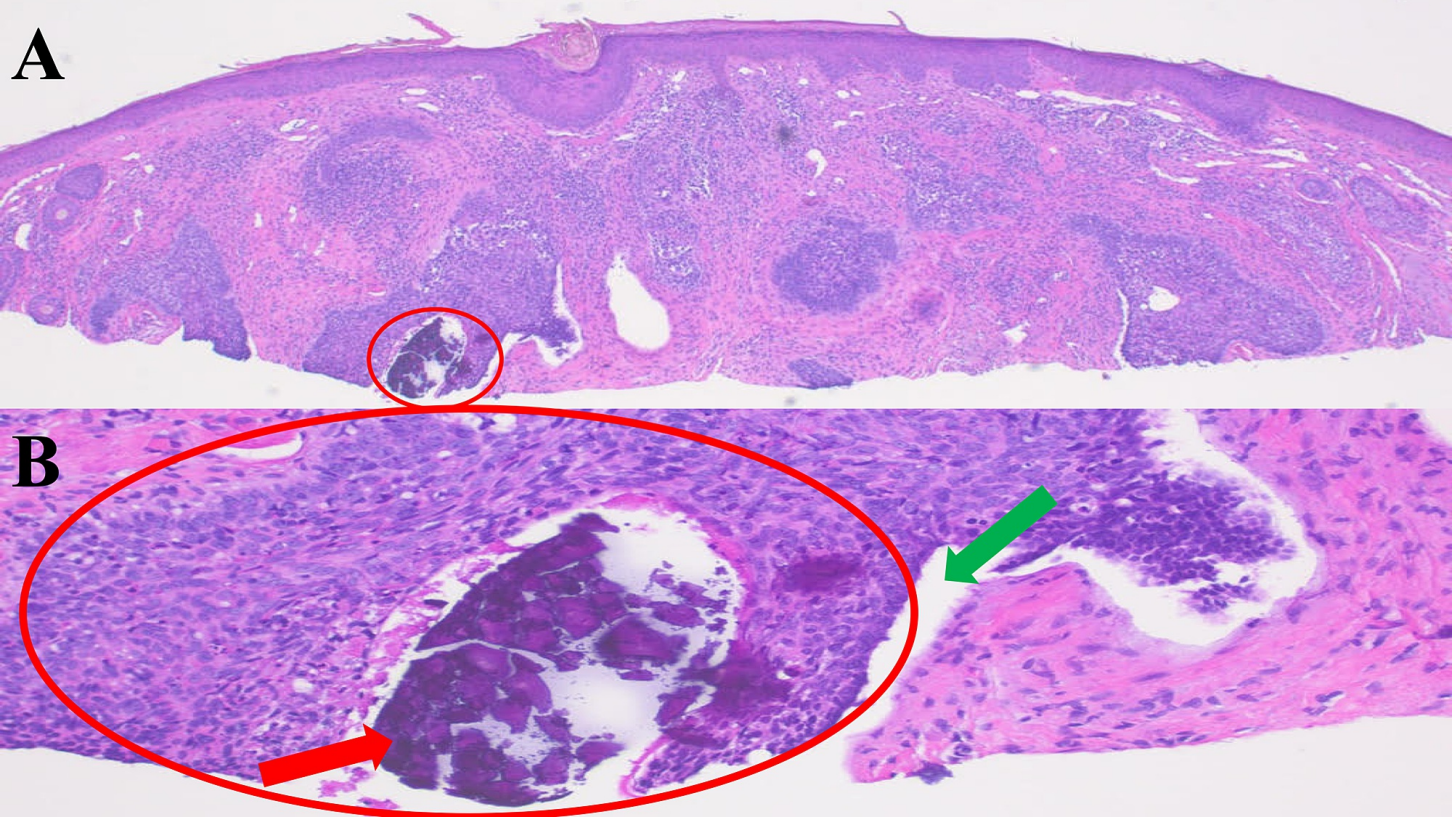
Histologic sections of a nodular basal cell carcinoma with one area of calcification The second half of the bisected tissue specimen shows one area of calcification within a nest of basal cell carcinoma (A). A closer view (B) shows a type 1 basal cell carcinoma-associated calcification (red arrow) with calcium in the aggregate of basaloid tumor cells (red circle). Stromal retraction (green arrow) is seen between the nest of tumor cells and the adjacent dermis.

Microscopic examination of the tissue specimen showed nodular aggregates of basaloid tumor cells with peripheral palisading of the tumor cells and retraction of the dermal stroma from the tumor aggregates. Calcification was also noted not only in the dermis and within a hair follicle adjacent to the tumor but also in the nest of tumor cells and in the necrotic debris associated with the basal cell carcinoma (Figure 2 and Figure 3).

Mohs surgery was performed; the nodular basal cell carcinoma with associated calcinosis cutis was removed in three stages. The size of the final defect was 2 centimeters by 1.4 centimeters; a full thickness skin graft taken from a posterior auricular site was used to fill the skin defect on his nose. There was no recurrence at 17 months follow-up.

## Discussion

Basal cell carcinoma is associated with several risk factors including arsenic exposure, genetic predisposition (such as with Gorlin syndrome and xeroderma pigmentosum), immunosuppression, light skin color, trauma, and ultraviolet (UV) radiation exposure. The main molecular pathogenesis is related to upregulation of the sonic hedgehog (SHH) pathway, in particular the GLI transcription factors. Inactivation of GLI transcription factors in mouse models led to basal cell carcinoma regression [4,5].

Gorlin syndrome, also called nevoid basal cell carcinoma syndrome, is an autosomal dominant condition. It manifests with basal cell carcinomas, skeletal abnormalities, pits in the palms of the hands or soles of the feet, and keratocysts in the jaw. Gorlin syndrome is associated with a 9q22.3 mutation involved in the SHH pathway, and this mutation has been identified in other individuals with basal cell carcinomas [2].

Second to upregulation in the SHH pathway, TP53 gene mutations are the most common genetic mutation associated with basal cell carcinoma. Mutations in the cyclin-dependent kinase inhibitor 2A (CDKN2A) gene and ras gene family have also been associated with basal cell carcinoma in a small number of cases. Studies with mouse models have identified follicular derivations for basal cell carcinoma as well [5].

Dystrophic calcification is the most common subtype of calcinosis cutis. It results from local or systemic injury to the skin. Etiologies associated with dystrophic calcification include connective tissue diseases (characterized by abnormal collagen or elastic fibers), inflammation, trauma, and tumors (including both benign and malignant neoplasms) [3].

Basal cell carcinoma can be associated with calcinosis cutis. It does not have a distinct clinical presentation; however, it may be observed as an incidental finding by the pathologist. Yet, there are very few studies in the literature on this topic.

The reported incidence of basal cell carcinoma with calcinosis cutis has been investigated by five studies (Table 1) [2,6-9]. The incidence of calcifying basal cell carcinoma ranged from 7% to 21% (median, 11%). If the data from the five studies are evaluated collectively, the incidence of basal cell carcinoma with calcinosis cutis is 14% (92 of 653 tumor specimens) [2,6-9].

**Table 1:** Incidence of calcinosis cutis in basal cell carcinoma BCC: Basal cell carcinoma; CC: calcinosis cutis; Ref: reference. ^a^The investigators evaluated 30 consecutive keratinizing basal cell carcinomas and 30 consecutive routine basal cell carcinomas. Calcification was observed in 30% (nine of 30) of the keratinizing basal cell carcinomas and 10% (three of 30) of the routine basal cell carcinomas [6]. ^b^Ninety four patients with a clinical diagnosis of basal cell carcinoma or “rule out basal cell carcinoma” were evaluated; 50 (53%) demonstrated basal cell carcinoma on deeper sections. The incidence of calcifying basal cell carcinoma was 10%; the deeper sections of five of the 50 specimens showed microcalcifications [9].

Incidence of BCC with CC	Number of BCC specimens with CC	Total number of BCC specimens	Location	Ref
21%	41	200	59% from head and neck (24/41); 37% from trunk (15/41); 5% from extremities (2/41)	[7]
20%^a^	12	60	Not mentioned	[6]
11%	27	243	70% from head and neck (58/83); 14.4% from trunk (12/83); 15.6% from upper limbs (13/83)	[2]
10%^b^	5	50	Not mentioned	[9]
7%	7	100	Not mentioned	[8]

Basal cell carcinoma with calcification is found in the head and neck in greater than 50% of patients. This is followed by lesions on the trunk and extremities (most commonly the upper limbs). One study of 200 patients found a greater incidence of basal cell carcinoma with calcification on the trunk (37%) compared with basal cell carcinoma without calcification on the trunk (19%) [2,7].

Basal cell carcinomas of the breast and areola are uncommon sites for this cutaneous neoplasm [4]. However, basal cell carcinoma with calcification can be identified in these areas incidentally when patients are undergoing mammographic examination (Table 2) [1,10-12]. This may be confused with ductal carcinoma in situ because calcification in the dermis of the breast tissue can falsely appear to be in the breast parenchyma [1,10-12].

**Table 2:** Basal cell carcinoma with calcification identified through mammographic examination BCC: Basal cell carcinoma; Ref: reference.

Age and gender	Location	BCC subtype	Clinical presentation	Treatment	Ref
53-year-old woman	Right breast	Not mentioned	No abnormal skin lesion visible	Not mentioned	[10]
64-year-old woman	Right breast	Nodular	No abnormal skin lesion visible	Complete excision	[11]
70-year-old man	Right areola	Nodular	Pink, scaly plaque with central depression	Circumareolar excision	[1]
82-year-old woman	Left nipple and areola	Not mentioned	Hyperpigmented and erosive papule	Nipple and areolar excision	[12]

Calcinosis cutis presents as homogenous purple deposits with hematoxylin and eosin staining of a specimen. Von Kassa staining may also be used to confirm the presence of calcium that appears as black or brown deposits. In a study of patients with either clinically diagnosed basal cell carcinoma or “rule out basal cell carcinoma,” 83% of patients (five of six individuals) with calcification on their initial nondiagnostic histological sections had basal cell carcinoma in deeper histological sections [3,9].

Similarly, subsequent investigators also discovered basal cell carcinoma after performing deeper sections of the paraffin-embedded tissue specimen when the initial sections only revealed calcification in the dermis without any accompanying tumor. Specifically, five patients with calcification free in the dermis in the superficial histologic sections of their tissue specimens were found to have a neoplasm after an investigation of deeper sections. Therefore, calcium in the initial superficial histological sections of a tissue specimen may be a diagnostic clue for the presence of basal cell carcinoma in deeper sections [2,9].

There are five categories of calcification in basal cell carcinoma. Slodkowska et al. identified four classifications of basal cell carcinoma with calcinosis cutis (Table 3) [2]. These include calcification in the tumor nest (type 1), calcification in keratinizing cysts within the carcinoma (type 2), calcification in necrotic debris within the neoplasm (type 3), and calcification in the stroma around the cancer or in the adjacent dermis (type 4).

**Table 3:** Classification and incidence of calcification in basal cell carcinoma BCC: Basal cell carcinoma; CR: current report; Ref: reference. ^a^Some of the basal cell carcinomas demonstrated more than one category of calcification.

Classification category	Definition	Incidence^a^	Number of specimens in classification/total specimens	Ref
Type 1	Calcium in BCC nests (in direct contact with neoplastic cells)	10%	9/83	[2]
Type 2	Calcified, keratinized cysts within BCC	58%	48/83	[2]
Type 3	Calcified, necrotic debris within BCC	14%	12/83	[2]
Type 4	Calcium in BCC stroma or adjacent dermis (not in contact with keratinocytes or basaloid cells)	53%	44/83	[2]
Type 5	Calcium in adnexal structures	Unknown	Unknown	[CR]

The most commonly occurring classification of basal cell carcinoma with calcinosis cutis is type 2 (calcified keratin pearls within basal cell carcinoma) with an incidence of 58% [2]. However, we observed another subtype of calcification in our patient. Therefore, we propose a fifth classification of basal cell carcinoma-associated calcification: calcinosis cutis in adnexal structures adjacent to the tumor.

Microscopic examination of basal cell carcinoma can reveal several different histological variants including - but not limited to - infiltrative/morpheaform, metatypical, micronodular, multifocal, nodular, and superficial subtypes. Among basal cell carcinomas, the nodular subtype was most commonly associated with calcification with an incidence of 37% (Table 4) [7]. Basal cell carcinoma with calcification was also found in more aggressive histologic subtypes (infiltrative and micronodular); the incidences were 29% and 27%, respectively [7].

**Table 4:** Incidence of calcification with regard to basal cell carcinoma histology subtypes BCC: Basal cell carcinoma; Ref: reference. ^a^In this study of 41 calcifying basal cell carcinomas, none were of the multifocal or superficial histologic subtypes [7].

BCC histologic subtype^a^	Incidence of BCC with calcification	Number of BCC histological subtype specimens/total calcifying BCC specimens	Ref
Nodular	37%	15/41	[7]
Infiltrative/morpheaform	29%	12/41	[7]
Micronodular	27%	11/41	[7]
Metatypical	7%	3/41	[7]

Calcium-binding proteins may play a role in the deposition of calcium in basal cell carcinomas. Skin calcium-binding protein is expressed in less differentiated keratinocytes that basal cell carcinomas can originate from. In contrast, squamous cell carcinomas arise from more differentiated keratinocytes with less expression of skin calcium-binding protein and less associated calcification [7].

Pilomatricomas, trichilemmal cysts, and trichoepitheliomas are benign growths associated with hair follicles. They can also display calcinosis cutis. Similar to the calcified keratin cysts in basal cell carcinoma (Slodkowska et al. type 2 classification of calcifying basal cell carcinoma), these benign tumors demonstrate follicular differentiation that may play a role in calcium deposition [2,7].

Basal cell carcinomas can also have follicular derivations. Calcium deposition is proposed to be associated with keratin; in particular, a calcium-binding protein present in keratin was thought to play a role in calcinosis cutis. However, in a comparison of basal cell carcinoma with and without calcification, no differences were found in hair keratins or matrix transcription factors; therefore, this hypothesis for basal cell carcinoma-associated calcinosis cutis is less likely [2,7].

The treatment of basal cell carcinoma is usually based on the histologic subtype, size, and location of the tumor. More aggressive tumors of the infiltrative, metatypical, and micronodular subtypes are best treated through excision with Mohs surgery. Less aggressive and smaller tumors may be treated with other modalities [13]. Our patient with a five-millimeter nodular, calcifying basal cell carcinoma on his nasal ala underwent Mohs micrographic surgery; his tumor required three stages to be cleared.

## Conclusions

The calculated incidence of calcinosis cutis in basal cell carcinoma is 14%; calcified, keratinized cysts within the basal cell carcinoma (type 2) and calcium in the stroma adjacent to the tumor (type 4) were the categories of calcification with the greatest incidence. Although nodular basal cell carcinomas are most commonly associated with calcium deposition, calcifying basal cell carcinoma frequently occurs with tumors that have a more aggressive histology subtype: infiltrative and micronodular basal cell carcinoma. The treatment of basal cell carcinoma with calcinosis cutis is based upon the histologic subtype, the size, and the location of the tumor. Our patient with a calcifying, nodular basal cell carcinoma was successfully treated with Mohs micrographic surgery.
